# Compliant Contact Force Control for Aerial Manipulator of Adaptive Neural Network-Based Robust Control

**DOI:** 10.3390/s24082556

**Published:** 2024-04-16

**Authors:** Qian Fang, Pengjun Mao

**Affiliations:** School of Mechanical and Electrical Engineering, Henan University of Science and Technology, Luoyang 471003, China; fangqian@haust.edu.cn

**Keywords:** aerial manipulator, sliding mode control, RBF neural network, impedance control

## Abstract

Aerial manipulators expand the application scenarios of manipulators into the air. To complete various operations, the contact force between the aerial manipulator and the target must be precisely controlled. In this study, we first established the mathematical models of the multirotor and the manipulator separately. Their mutual influence is regarded as each other’s disturbance, and the overall linkage mechanism is established through analysis. Then, a robust sliding mode control strategy is developed for accurate trajectory tracking. The controller is derived from Lyapunov theory, which can ensure the stability of the closed-loop system. To compensate for the effect of system uncertainty, an adaptive radial basis function neural network is devised to approximate the part of the controller containing the model information. In addition, an impedance controller is designed to convert force control into position control to make the manipulator contact with the target compliantly. Finally, the simulation and experimental results indicate that the proposed method can guarantee the accuracy of the contact force and has good robustness.

## 1. Introduction

In recent years, robot manipulators have been widely used in many fields, such as manufacturing [1], assembly [2], grinding [3], and drilling [4]. However, these robotic arms are almost always mounted on fixed bases, significantly limiting their working space. In order to expand the operating space, installing the manipulator on a non-fixed platform for operation has become a hot topic in current research. As a combination of aerial platform and manipulator, the aerial manipulator has better maneuverability in three-dimensional space and flexibility to operate. It has consequential value in multiple domains like national defense, rescue, and scientific research and has recently attracted more and more attention from researchers.

The Unmanned Aerial Vehicle (UAV) platform and the manipulator are both highly nonlinear systems, and their combination makes the entire system more complex. In addition, external interference and internal uncertainty make it more difficult to control the system accurately. Some researchers treat the UAV and the manipulator as an entirety, establish a unified model, and design a set of controllers to control all system states directly [5,6,7]. However, the system developed by this method has high dimensions. For embedded systems such as aerial manipulators, their computing resources are limited, making it hard to realize real-time calculation of the system state. Especially when the load of the manipulator changes or contact force occurs with the external environment, the accuracy of the coupling model established offline is incredibly reduced, which will seriously affect the controller’s performance. Therefore, many researchers regard the UAV platform and the manipulator as two separate subsystems and the interaction between them as mutual disturbances, which dramatically reduces the dimensionality of the system model. By designing controllers for each subsystem, the controller’s performance can be effectively improved [8,9,10,11,12]. These studies use multi-rotors as motion platforms to drive manipulators to complete operating tasks. However, the body coordinate system of the multi-rotor changes constantly when it is moving, which causes the coordinate system of the manipulator to change with the body coordinate system constantly. Continuous coordinate transformation will seriously affect the accuracy of control. Therefore, a more stable contact method and robust controller are needed to achieve high-precision contact force operations.

In this paper, we propose an adaptive robust control strategy based on impedance control for the aerial manipulator to exert the contact force accurately. The main contributions can be summarized as follows:

1. By discussing the characteristics of aerial operations, a new operating mode for aerial manipulators is designed, in which the UAV platform only provides a fixed fulcrum in the air, and the manipulator works as the fixed manipulator.
2. A nonsingular global fast terminal sliding mode control (NGFTSMC) method is proposed, which can quickly converge the state error in a limited time to achieve accurate trajectory tracking of UAVs and robotic arms. Furthermore, to make the system resistant to the model’s uncertainty, RBFNN is used to estimate the part of the controller that contains the model information.
3. A double-layer control structure is designed to achieve compliant contact between the manipulator and the target and reduce the impact of contact force on the stability of the aerial manipulation system. The outer layer impedance control converts the expected contact force into the desired position trajectory, and the inner layer position control realizes the trajectory tracking of the desired position.

The rest of this article is organized as follows. Section 2 provides an overview of related work. Section 3 describes the dynamics model of the UAV platform and the manipulator, respectively. Section 4 designs robust sliding mode controllers based on adaptive RBFNN for both subsystems and an impedance controller for the compliant contact of the aerial manipulator. The simulation and experimental results and analysis are shown in Section 5 and Section 6. Finally, Section 7 gives the conclusion.

## 2. Related Work

The fixed base in the air dramatically expands the operating range of the manipulator, but the key to achieving an aerial fixed base is precise trajectory tracking. The multirotor is an underactuated, strongly coupled nonlinear system, and its sensitivity to internal uncertainty and external disturbances is essential to its stability. Moreover, the manipulator’s movement and the target’s reaction force also seriously affect the stability of the multirotor. Designing effectively robust controllers to fix these problems poses a significant challenge to researchers.

Many studies have shown that the sliding mode controller is one of the most effectively robust control techniques. Almakhles DJ designed a double-loop control structure and proposed a controller with integral sliding mode and backstepping sliding mode methods to ensure the position trajectory tracking capability under uncertainties [13]. Labbadi M improved proportional integral differential sliding mode control with the super-twist algorithm, providing good robustness against the time-varying external disturbances, solving the chattering issue, and avoiding discontinuousness of input signals [14]. Wang X proposed a sensor-based Incremental Nonlinear Dynamic Inversion Sliding Mode Control driven by Sliding Mode Disturbance Observers, which can simultaneously reduce the model dependency of the controller and the uncertainties in the closed-loop system [15]. However, the traditional sliding mode control method cannot guarantee the rapid convergence of trajectory tracking within a limited time, which is an enormous disadvantage for real-time systems. Therefore, terminal sliding mode control methods were proposed to solve this problem. Labbadi M designed a double-loop control strategy, where the outer layer utilizes a robust adaptive back-stepping controller to control the position, and the inner layer uses a controller that combined back-stepping and fast terminal sliding modes to control the attitude [16]. Nekoukar V suggested a new robust flight control system consisting of an adaptive fuzzy terminal sliding mode controller and two proportional-derivative controllers to guarantee flight stability and efficient tracking of pre-defined paths [17]. Tripathi VK designed a finite-time super-twisting sliding mode control scheme to assure finite-time convergence of tracking error with chattering attenuation and developed a high-order sliding mode observer to determine unknown bounded lumped disturbances acting on the quadrotor [18]. In [19], he also proposed an adaptive fast terminal sliding-mode controller with a power ratio proportional reaching law, which can track the position and altitude of a quadrotor with parametric uncertainties and external disturbances. Razmjooei H presented a time-varying chattering-free disturbance observer-based position tracking control law of serial robotic manipulators to track a reference signal in a finite time and employ a positive-increasing function associated with the control/observer objectives to improve the control performance [20].

The design of the above controllers depends on the system model. Nevertheless, the parameters of the system model often change during actual operation, which will seriously deteriorate the performance of the controller. The Radial Basis Function Neural Network (RBFNN) is a special type of neural network structure that can design an adaptive rate based on the Lyapunov stability theorem. It has strong self-learning and nonlinear mapping abilities and can approximate any nonlinear function. Therefore, many controllers are designed based on RBFNN to eliminate their dependence on the system model and compensate for uncertain signals to obtain better trajectory tracking effects. Luo H developed a robust double-loop control scheme for quadrotor speed and attitude tracking, where the outer loop was designed with a coupling controller to ensure velocity tracking based on the stability of altitude tracking control and the inner loop to control the attitude by utilizing the RBFNN to compensate for model disturbance uncertainty [21]. Tao M proposed a singularity-free terminal sliding mode control scheme improved by RBFNN and Extended State Observer (ESO) that does not require prior knowledge about unknown nonlinear uncertainties and external disturbances for quadrotor UAVs [22]. Zhang Q proposed a terminal sliding mode attitude controller based on the RBFNN uncertainty compensator and optimized it via a particle swarm intelligence algorithm with two fitness functions, solving the non-unique actuator action caused by over-actuation of the tilt-rotor quadcopter [23]. Huang S proposed a non-singular fast terminal sliding mode controller for trajectory tracking control of the quadrotor, with a disturbance observer to estimate the external interference and a neural network approximator to develop an online estimate of the model uncertainty [24].

The manipulator is the operational part of the aerial manipulation system. Environmental constraints restrict the movement of the manipulator and generate contact forces. To reduce the impact of the target’s reaction force on the entire aerial manipulation system, the contact between the manipulator and the target should be compliant. Compliance control has been widely used in robotics, such as welding [25], polishing [26], and human–computer interaction [27]. Traditional compliance control methods include force/position hybrid control [28] and impedance control [29]. In actual environments, the contact between the manipulator and the target has problems of low absolute accuracy and lack of real pose information, which makes force/position hybrid control hard to achieve. Nonetheless, the impedance control theory applies the manipulator’s motion trajectory and contact force to a dynamic framework, so the contact force can be controlled by following the position trajectory. Therefore, the manipulator also needs to design a high-precision trajectory-tracking controller.

The relevant theories for designing trajectory-tracking controllers for quadrotors are also widely used in manipulators. Tran DT proposed a non-singular fast terminal sliding mode controller for manipulator position tracking and used adaptive RBFNN to approximate and eliminate uncertainties [30]. Zhang W utilized the fractional-order method in the proposed non-singular fast terminal sliding mode controller to improve the tracking performance of the controller and designed RBFNN to approximate the unknown nonlinear function of the system to accomplish model-free control [31]. Jie W proposed a fast fractional-order terminal sliding mode controller with a new perturbation estimator that applied the data-driven method RBFNN to compensate for the estimation error in the conventional sliding perturbation observer to improve the tracking accuracy and reduce the chattering [32]. Kim SJ proposed an adaptive robust RBFNN non-singular terminal sliding mode controller to reduce swinging in the snake robot’s head where the RBFNN compensates for interference and an adaptive robust term to make up for the shortcomings of neural network control to eliminate system chattering [33].

This study designed a new contact force control strategy based on the aerial manipulation system composed of a multirotor UAV and a multi-joint manipulator. First, to provide a fixed aerial platform for the manipulator, a non-singular global fast terminal sliding mode controller based on RBFNN was designed for accurate trajectory tracking. Then, a double-loop control scheme was designed for the compliant contact between the manipulator and the target. The outer loop used a position-based impedance controller to generate the desired trajectory, and the inner loop proposed a trajectory-tracking control algorithm based on the design concept of the UAV platform controller.

## 3. System Modeling

**Figure 1:**
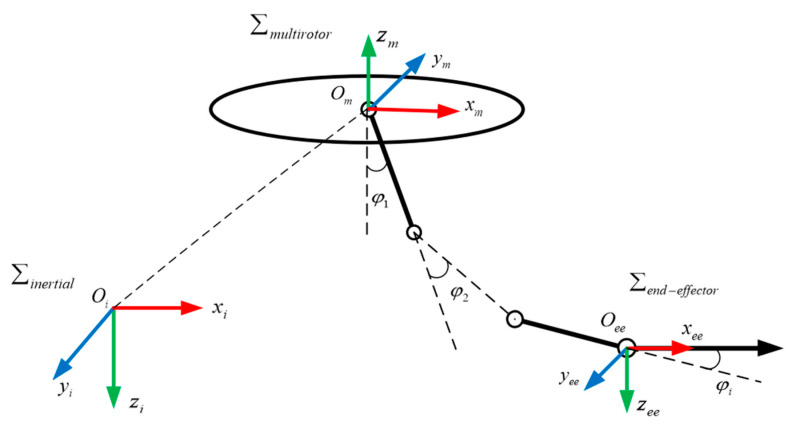
Aerial manipulation system structure diagram.

The aerial manipulation system consists of a multirotor UAV and an n-link manipulator. The system structure is shown in Figure 1.

In Figure 1, $\Sigma_{inertial}$ represents the inertial coordinate system with the origin at $O_{i}$, $\Sigma_{multirotor}$ represents the multirotor body coordinate system with the origin at $O_{m}$, $\Sigma_{end-effector}$ represents the end-effector coordinate system with the origin at $O_{ee}$, and $\varphi_{i}$ is the angle of rotation of the i-th link manipulator.

To simplify the system structure, we assume:

(1) The body of the multirotor and manipulator is rigid, and the structure is symmetrical.

(2) The masses of the multirotor and manipulator are evenly distributed, and the centers of mass coincide with the geometric centers.

Combining the multirotor and manipulator increases the control dimension of the entire system. For embedded systems such as aerial manipulators, real-time high-dimensional matrix transformation calculation is hard to achieve. Therefore, this study regards the multirotor and the manipulator as two independent subsystems. The multirotor subsystem is only used as a fixed aerial platform, while the manipulator subsystem is only used as the operating tool. The forces between them are regarded as mutual disturbances. Based on this setting, we built the models of two subsystems separately.

### 3.1. Multirotor Platform Modeling

Assuming that the multirotor is rigid, its dynamic equation can be described using the Newton–Euler formula as follows:

(1) $$\left\{\begin{array}{l} m\ddot{P}+mge_{3}=u_{P}+\tau_{P}\\ J\ddot{\Phi }+C\left(\Phi ,\dot{\Phi }\right)\dot{\Phi }=u_{\Phi }+\tau_{\Phi } \end{array}\right.$$

where $P={\left[x,y,z\right]}^{T}$ and $\Phi ={\left[\phi ,\theta ,\psi \right]}^{T}$ represent the position and Euler angle of the multirotor in the inertial coordinate system, respectively; m is the mass of the entire aerial manipulator; g is the acceleration of gravity; $e_{3}={\left[0,0,1\right]}^{T}$ is the unit vector along the $O_{i}z_{i}$ axis in the inertial coordinate system; J and C represent the inertia and coriolis matrix in the inertial coordinate system; $u_{P}$ and $\tau_{P}$ represent the thrust force and disturbance generated by the multirotor in the translational direction; and $u_{\Phi }$ and $\tau_{\Phi }$ represent the torque and disturbance received in the rotational direction.

The rotation matrix $R_{m}^{i}$ from the multirotor body coordinate system $\Sigma_{multirotor}$ to the inertial coordinate system $\Sigma_{inertial}$ can be expressed as

(2) $$R_{m}^{i}=\left[\begin{array}{ccc} \cos\theta \cos\psi & \sin\phi \sin\theta \cos\psi -\cos\phi \sin\psi & \cos\phi \sin\theta \cos\psi +\sin\phi \sin\psi \\ \cos\theta \sin\psi & \sin\phi \sin\theta \sin\psi +\cos\phi \cos\psi & \cos\phi \sin\theta \sin\psi -\sin\phi \cos\psi \\ -\sin\theta & \sin\phi \cos\theta & \cos\phi \cos\theta \end{array}\right]$$

The movement process of aerial manipulators aiming at accomplishing precise contact operations is usually performed within a restricted range. This type of operation requires high stability of the multirotor platform but low requirements for its maneuverability. Therefore, when the multirotor UAV runs at a low speed and small angle, Equation (1) can be rewritten through simplification and decoupling as

(3) $$\left\{\begin{array}{l} \ddot{x}=\left(cos\phi sin\theta cos\psi +sin\phi sin\psi \right)\frac{u_{1}}{m}+d_{x}\\ \ddot{y}=\left(cos\phi sin\theta sin\psi -sin\phi cos\psi \right)\frac{u_{1}}{m}+d_{y}\\ \ddot{z}=cos\phi cos\theta \frac{u_{1}}{m}-g+d_{z} \end{array}\right.$$

(4) $$\left\{\begin{array}{l} \ddot{\phi }=\left(\frac{I_{yy}-I_{zz}}{I_{xx}}\right)\dot{\theta }\dot{\psi }-\frac{J}{I_{xx}}\dot{\theta }\Omega +\frac{u_{2}}{I_{xx}}+d_{\phi }\\ \ddot{\theta }=\left(\frac{I_{zz}-I_{xx}}{I_{yy}}\right)\dot{\phi }\dot{\psi }-\frac{J}{I_{yy}}\dot{\phi }\Omega +\frac{u_{3}}{I_{yy}}+d_{\theta }\\ \ddot{\psi }=\left(\frac{I_{xx}-I_{yy}}{I_{zz}}\right)\dot{\phi }\dot{\theta }+\frac{u_{4}}{I_{zz}}+d_{\psi } \end{array}\right.$$

where $I_{i}(i=xx,yy,zz)$ represents the inertia matrix of the aerial manipulator; $J_{m}$ represents the rotational inertia of the multirotor motor; Ω represents the total remaining speed of the multirotor motor; $u_{i}(i=1,2,3,4)$ is the control input of the multirotor; and $d_{i}(i=x,y,z,\phi ,\theta ,\psi )$ represents the lumped disturbance caused by the internal uncertainty, the external environment, and the action of the manipulator.

### 3.2. Manipulator Modeling

According to Newton–Euler’s theorem, the Cartesian dynamics equation of an n-DOF (Degree of Freedom) rigid robot manipulator system can be expressed as

(5) $$B\left(\varphi \right)\ddot{\varphi }+H\left(\varphi ,\dot{\varphi }\right)\dot{\varphi }+V\left(\varphi \right)+T_{c}+T_{d}=T$$

where $\varphi ={\left[\varphi_{1}\;\varphi_{2}\;\cdots \;\varphi_{i}\right]}^{T}$ is the joint angular vector of the *i*-link manipulator, $\dot{\varphi },\ddot{\varphi }$ represent the joint angular velocity vector and acceleration vector of the manipulator, $B\left(\varphi \right)$ is the symmetric positive definite inertia matrix, $H\left(\varphi ,\dot{\varphi }\right)$ is the centrifugal force and coriolis force matrix, $V\left(\varphi \right)$ is the gravity matrix, T is the joint torque control input vector, $T_{c}$ is the torque vector of the interaction between the manipulator and the target, and $T_{d}$ is the lumped disturbance of the manipulator.

Let η be the position vector of the end effector in the task space. Joint space can be mapped to task space through forward kinematics as follows:

(6) $$\eta =Map\left(\varphi \right)$$

where $Map\left(\varphi \right)$ is the mapping relationship from the joint space to the task space. Therefore, the relationship between the velocity in joint space and the velocity in task space can be described as

(7) $$\dot{\eta }=J\left(\varphi \right)\dot{\varphi }$$

where the Jacobian matrix J(φ) represents the relationship between the virtual end effector speed and the virtual joint speed. By differentiating Equation (7), the acceleration term of the end effector is determined as

(8) $$\ddot{\eta }=J\left(\varphi \right)\ddot{\varphi }+\dot{J}\left(\varphi \right)\dot{\varphi }$$

Then, the dynamics of the manipulator in the task space can be expressed as

(9) $$\begin{array}{c} B_{\eta }\left(\varphi \right)\ddot{\eta }+H_{\eta }\left(\varphi ,\dot{\varphi }\right)\dot{\eta }+V_{\eta }\left(\varphi \right)+F_{c}+F_{d}=F_{\eta }\\ B_{\eta }\left(\varphi \right)=J^{-T}(\varphi )B\left(\varphi \right)J^{-1}(\varphi )\\ H_{\eta }\left(\varphi ,\dot{\varphi }\right)=J^{-T}(\varphi )\left(H\left(\varphi ,\dot{\varphi }\right)-B\left(\varphi \right)J^{-1}(\varphi )\dot{J}(\varphi )\right)J^{-1}(\varphi )\\ V_{\eta }\left(\varphi \right)=J^{-T}(\varphi )V\left(\varphi \right)\\ F_{\eta }=J^{-T}(\varphi )T\\ F_{c}={J^{-T}(\varphi )T}_{c}\\ F_{d}={J^{-T}(\varphi )T}_{d} \end{array}$$

### 3.3. Aerial Platform Attitude Compensation

The contact force between the manipulator and the target is generated by the torque of the manipulator motor, and the corresponding counter-torque acts on the aerial platform and is offset by the thrust generated by the multirotor. Since the lift of the propeller is the only source of power for the aerial manipulator in the air, it can be considered that the contact force is generated by the thrust of the multirotor. Therefore, in order for the manipulator to exert a stable contact force on the target, the multirotor platform should always stay hovering. According to Equation (3), disregarding the influence of disturbance, the equilibrium relationship of contact force exerted in the hovering state can be obtained as

(10) $$\left\{\begin{array}{l} \left(cos\phi sin\theta cos\psi +sin\phi sin\psi \right)u_{1}-F_{c-x}=0\\ \left(cos\phi sin\theta sin\psi -sin\phi cos\psi \right)u_{1}-F_{c-y}=0\\ cos\phi cos\theta u_{1}-mg-F_{c-z}=0 \end{array}\right.$$

In this study, the manipulator generates contact force with the vertical target in a point contact mode, so $F_{c-y}=0$ and $F_{c-z}=0$. We can set the yaw angle ψ=0; then according to Equation (10), the contact force $F_{c-x}$ can be calculated as

(11) $$F_{c-x}=mg\tan\theta$$

It can be seen that $F_{c-x}$ is determined by the gravity mg and pitch angle θ of the quadrotor. Since the gravity of the aerial manipulator is constant, the value of $F_{c-x}$ corresponds to the pitch angle θ one-to-one. Therefore, when the desired contact force is $F_{desired}$, the desired pitch angle $\theta_{desired}$ of the multirotor can be calculated as

(12) $$\theta_{desired}=\arctan\left(\frac{F_{desired}}{mg}\right)$$

**Figure 2:**
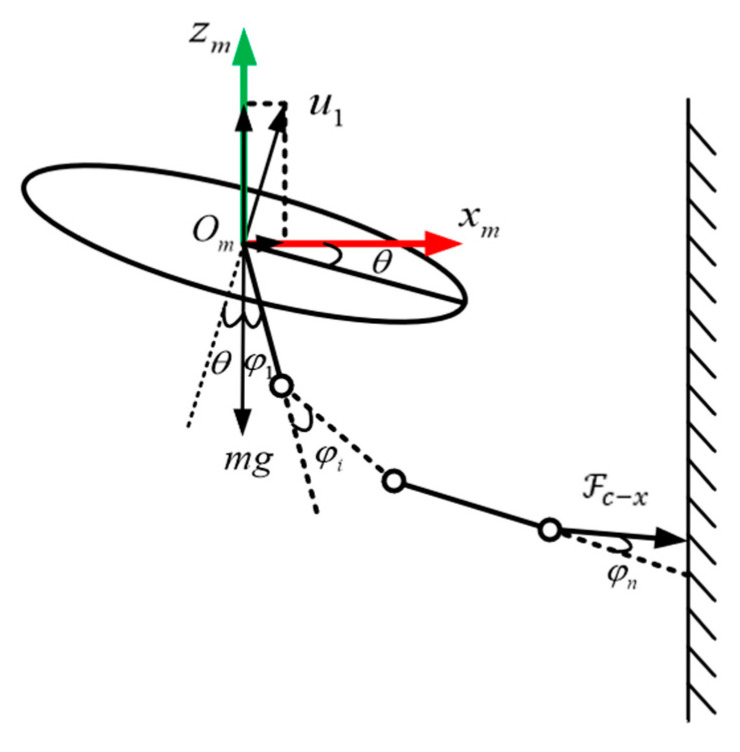
Analysis of contact between aerial manipulator and target.

As the aerial platform for the manipulator, changes in the attitude of the multirotor will change the relationship between the manipulator coordinate system and the inertial coordinate system. To prevent the kinematic models of the two subsystems from interfering with each other, the changes in the attitude of the multirotor should be compensated by the corresponding angle of the manipulator joint rotation so that the multirotor can be regarded as a fixed platform in the air. The above analysis is illustrated in Figure 2.

## 4. Controller Design and Stability Analysis

In this part, we first designed a robust trajectory-tracking controller for the multirotor and used RBFNN to eliminate the controller’s dependence on the system model. Then, to achieve the compliance contact of the manipulator, an impedance position controller was devised to obtain the manipulator’s desired trajectory. Finally, bringing in the multirotor controller’s design theories, the manipulator’s trajectory-tracking controller was developed.

### 4.1. Multirotor Platform Controller Design

Taking the height controller as an example, the height error is defined as

(13) $$e=z-z_{d}$$

where $z_{d}$ is the desired height. We can design the nonsingular fast terminal sliding mode surface as

(14) $$s=\dot{e}+\alpha_{1}e+\beta_{1}{\left|e\right|}^{\mu_{1}/v_{1}}sgn\left(e\right)$$

where $\alpha_{1}>0$, $\beta_{1}>0$, $\mu_{1}$ and $v_{1}$ are positive odd numbers and satisfy $\mu_{1}<v_{1}$. sgn(e) is the switching function of the error.

(15) $$sgn\left(e\right)=\left\{\begin{array}{c} 1,e>0\\ 0,e=0\\ -1,e<0 \end{array}\right.$$

Taking the time derivative of Equation (14),

(16) $$\dot{s}=\ddot{e}+\alpha_{1}\dot{e}+\beta_{1}\frac{\mu_{1}}{v_{1}}{\left|e\right|}^{\left(\mu_{1}-v_{1}\right)/v_{1}}\dot{e}sgn\left(e\right)$$

Equation (11) can be rewritten as follows by substituting Equation (3):

(17) $$\dot{s}=\frac{cos\phi cos\theta }{m}u_{1}-g+d_{z}-{\ddot{z}}_{d}+\alpha_{1}\dot{e}+\beta_{1}\frac{\mu_{1}}{v_{1}}{\left|e\right|}^{\left(\mu_{1}-v_{1}\right)/v_{1}}\dot{e}sgn\left(e\right)$$

In order to promote the system state to reach the sliding surface during the entire approximation process quickly, the fast arrival law is proposed as

(18) $$\dot{s}=-\alpha_{2}{\left|s\right|}^{\mu_{2}}sgn\left(s\right)-\beta_{2}{\left|s\right|}^{v_{2}}sgn(s)$$

where $\alpha_{2}>0$, $\beta_{2}>0$, $0<\mu_{2}<1$ and $v_{2}>1$. According to Equations (17) and (18), the controller $u_{1}$ is designed as

(19) $$\begin{array}{c} u_{1}=\frac{m}{cos\phi cos\theta }({\ddot{z}}_{d}+g-\alpha_{1}\dot{e}-\beta_{1}\frac{\mu_{1}}{v_{1}}{\left|e\right|}^{\left(\mu_{1}-v_{1}\right)/v_{1}}\dot{e}sgn\left(e\right)\\ -\alpha_{2}{\left|s\right|}^{\mu_{2}}sgn\left(s\right)-\beta_{2}{\left|s\right|}^{v_{2}}sgn(s)-D_{z}sgn\left(s\right)) \end{array}$$

where $D_{z}>\left|d_{z}\right|$ is the robust term of the controller; its function is to overcome the influence of external disturbance on the system trajectory and guide it to move to the sliding mode surface. We can define the Lyapunov function as

(20) $$V_{1}=\frac{1}{2}s^{2}$$

Taking the time derivative of Equation (20) and substituting Equation (17)

(21) $${\dot{V}}_{1}=s\dot{s}=s\left(\frac{cos\phi cos\theta }{m}u_{1}-g+d_{z}-{\ddot{z}}_{d}+\alpha_{1}\dot{e}+\beta_{1}\frac{\mu_{1}}{v_{1}}{\left|e\right|}^{\left(\mu_{1}-v_{1}\right)/v_{1}}\dot{e}sgn\left(e\right)\right)$$

Substituting Equation (19) into Equation (21), ${\dot{V}}_{1}$ can be deduced as

(22) $$\begin{array}{c} {\dot{V}}_{1}=-\alpha_{2}{\left|s\right|}^{\mu_{2}+1}-\beta_{2}{\left|s\right|}^{v_{2}+1}-sD_{z}sgn\left(s\right)+d_{z}s\\ =-\alpha_{2}{\left|s\right|}^{\mu_{2}+1}-\beta_{2}{\left|s\right|}^{v_{2}+1}-\left(D_{z}\left|s\right|-d_{z}s\right) \end{array}$$

When $D_{z}\geq \left|d_{z}\right|$, ${\dot{V}}_{1}\leq 0$, the system converges stably. According to the sliding mode control theory, the equivalent part of the designed controller is

(23) $$u_{eq}=\frac{m}{cos\phi cos\theta }\left({\ddot{z}}_{d}+g-\alpha_{1}\dot{e}-\beta_{1}\frac{\mu_{1}}{v_{1}}{\left|e\right|}^{\left(\mu_{1}-v_{1}\right)/v_{1}}\dot{e}sgn\left(e\right)\right)$$

The equivalent controller is related to the system model. In order to eliminate the impact of model uncertainty on the controller, we introduce the RBF neural network to estimate the equivalent controller. We can define the input of the RBF neural network as $X={\left(e_{z}\;{\dot{e}}_{z}\;z_{d}\;{\dot{z}}_{d}\;{\ddot{z}}_{d}\right)}^{T}$, and the equivalent control in Equation (23) is the ideal RBF neural network output, which can be expressed as

(24) $$u_{eq}=W^{T}h\left(X\right)+\varepsilon$$

where W is the ideal weight of the neural network, ε is a small positive real number that represents the approximation error of the neural network to the nonlinear uncertain function, and $h\left(X\right)$ is the Gaussian function that nonlinear mapping of the neural network, which can be expressed as

(25) $$h_{i}\left(X\right)=exp\frac{-{\left\|X-c_{j}\right\|}^{2}}{b_{j}^{2}},j=1,2,\cdots ,ℵ$$

where $c_{j}$ and $b_{j}$ are the center value and width of the Gaussian function, and ℵ is the number of neurons in the hidden layer of the network. We can let ${\hat{u}}_{eq}$ be the approximation output of the RBF neural network to the equivalent controller $u_{eq}$:

(26) $${\hat{u}}_{eq}={\hat{W}}^{T}h\left(X\right)$$

where $\hat{W}$ is the approximation weight of the RBF neural network. We can define the error induced by the neural network estimation as

(27) $${\tilde{u}}_{eq}=u_{eq}-{\hat{u}}_{eq}=W^{T}h\left(X\right)+\varepsilon -{\hat{W}}^{T}h\left(X\right)={\tilde{W}}^{T}h\left(X\right)+\varepsilon$$

where ${\tilde{W}}^{T}=W^{T}-{\hat{W}}^{T}$. Redesign $u_{1}$ as

(28) $$u_{1}={\hat{u}}_{eq}-\frac{m}{cos\phi cos\theta }(\alpha_{2}{\left|s\right|}^{\mu_{2}}sgn\left(s\right)+\beta_{2}{\left|s\right|}^{v_{2}}sgn\left(s\right))-\frac{m}{cos\phi cos\theta }{(D}_{z}+\Upsilon_{z})sgn(s)$$

where $\Upsilon_{z}>0$. Equation (17) can be rewritten as

(29) $$\begin{array}{cc} \dot{s}=\frac{cos\phi cos\theta }{m}u_{1} & -\frac{cos\phi cos\theta }{m}u_{eq}+d_{z}\\ & =\frac{cos\phi cos\theta }{m}\left({\hat{u}}_{eq}-u_{eq}\right)-\alpha_{2}{\left|s\right|}^{\mu_{2}}sgn\left(s\right)-\beta_{2}{\left|s\right|}^{v_{2}}sgn(s)-{(D}_{z}+\Upsilon_{z})sgn(s)+d_{z}\\ & =-\frac{cos\phi cos\theta }{m}\left({\tilde{W}}^{T}h\left(X\right)+\varepsilon \right)-\alpha_{2}{\left|s\right|}^{\mu_{2}}sgn\left(s\right)-\beta_{2}{\left|s\right|}^{v_{2}}sgn(s)-{(D}_{z}+\Upsilon_{z})sgn(s)+d_{z} \end{array}$$

We can design the Lyapunov function as

(30) $$V_{2}=\frac{1}{2}s^{2}+\frac{1}{2\xi }{\tilde{W}}^{T}\tilde{W}$$

Equation (30) can be rewritten as follows by taking the time derivative:

(31) $$\begin{array}{cc} {\dot{V}}_{2}=s\dot{s}+\frac{1}{\xi }{\tilde{W}}^{T}\dot{\tilde{W}} & =-\alpha_{2}{\left|s\right|}^{\mu_{2}+1}-\beta_{2}{\left|s\right|}^{v_{2}+1}-s{(D}_{z}+\Upsilon_{z})sgn(s)+d_{z}s-\frac{cos\phi cos\theta }{m}\varepsilon s-\frac{cos\phi cos\theta }{m}s{\tilde{W}}^{T}h\left(X\right)+\frac{1}{\xi }{\tilde{W}}^{T}\dot{\tilde{W}}\\ & =-\alpha_{2}{\left|s\right|}^{\mu_{2}+1}-\beta_{2}{\left|s\right|}^{v_{2}+1}-\left(D_{z}\left\|s\right\|-d_{z}s\right)-\left(\Upsilon_{z}\left\|s\right\|+\frac{cos\phi cos\theta }{m}\varepsilon s\right)-{\tilde{W}}^{T}(\frac{1}{\xi }\dot{\hat{W}}+\frac{cos\phi cos\theta }{m}sh\left(X\right)) \end{array}$$

We can define the adaptive law as

(32) $$\dot{\hat{W}}=-\frac{cos\phi cos\theta }{m}\xi sh\left(X\right)$$

**Figure 3:**
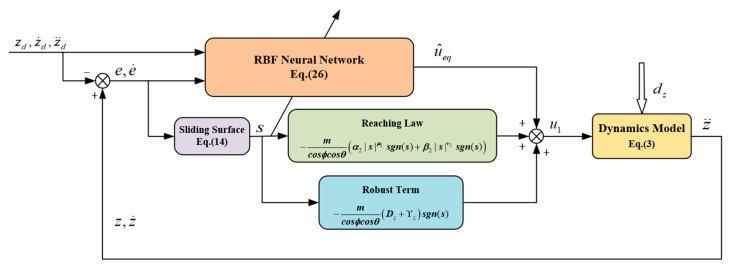
The structure of the quadrotor height controller.

When $\Upsilon_{z}\geq \left|\frac{cos\phi cos\theta }{m}\varepsilon \right|$, ${\dot{V}}_{2}\leq 0$, the system is stable. The structure of the proposed nonsingular global fast terminal sliding mode controller based on RBF neural network is shown in Figure 3.

Controllers for other system states can be designed according to the same technique. However, the horizontal states {x,y} and Euler angles {ϕ,θ} are coupled, and they need to be decoupled by set virtual control variables before the controller can be designed.

### 4.2. Manipulator Controller Design

The multirotor platform’s high-precision trajectory tracking performance can provide a steady aerial base. However, the reaction force generated by the contact between the manipulator and the target will significantly impact the stability of the entire system, making the contact force uncontrollable. In order to make the contact between the manipulator and the target compliant and the contact force change smoothly, this paper proposes a double-loop control structure. The outer loop is position-based impedance control, which converts the desired contact force into the desired position trajectory. The inner loop is a trajectory-tracking controller that accurately tracks the position trajectory generated by the outer loop.

#### 4.2.1. Outer Loop Position-Based Impedance Control

The contact procedure of the manipulator and the target is divided into two stages. The first stage involves the manipulator approaching the target, which is free space control. The desired position is the target position, where the flying robotic arm comes into contact with the environment. The second stage is manipulator contact with the target, which is restricted space control, and the desired position is generated by the desired force. To reduce contact impact on the system, an additional impedance control loop is added beside the position control. Based on the impedance relationship model, impedance control has the advantage of having force and position in the same framework, and the relationship between them can be adjusted by changing the impedance parameters. Commonly, the mathematical model of the impedance relationship can be expressed in terms of a second-order differential equation.

(33) $$M\ddot{E}+B\dot{E}+KE=F_{t}$$

where M, B, K are the required inertia, damping and stiffness matrices, respectively, and $F_{t}$ is the contact force that is exerted by the manipulator on the target measured by the sensor. E is the error between the planned reference trajectory $\eta_{r}$ and the expected trajectory $\eta_{d}$ calculated based on the contact force, which can be obtained from the Laplace transform of Equation (33) as

(34) $$E\left(s\right)=\frac{1}{Ms^{2}+Bs+K}F_{t}\left(s\right)$$

During the two stages of the contact process, the expected trajectory of the inner position control loop is

(35) $$\eta_{d}=\left\{\begin{array}{ll} \eta_{t} & \left(free\;space\right)\\ \eta_{r}-E & \left(contact\;space\right) \end{array}\right.$$

The reference trajectory $\eta_{r}$ is determined by the position and parameters of the manipulator, the impedance controller parameters, and the desired contact force $F_{\Xi }$. Equation (33) can be rewritten as

(36) $$M\left({\ddot{\eta }}_{r}-\ddot{\eta }\right)+B\left({\dot{\eta }}_{r}-\dot{\eta }\right)+K\left(\eta_{r}-\eta \right)=F_{\Xi }$$

The contact force is usually determined by the stiffness and damping parameters of the target, and it can be described by the following second-order nonlinear function.

(37) $$F_{\Xi }=K_{t}\left(\eta -\eta_{t}\right)+B_{t}\dot{\eta }$$

where $K_{t}$ and $B_{t}$ are the target’s diagonal positive definite stiffness matrices and damping matrices, respectively. Taking the contact force in a single direction as an example, Equations (36) and (37) can be rewritten as

(38) $$\left\{\begin{array}{l} m\left({\ddot{\delta }}_{r}-\ddot{\delta }\right)+b\left({\dot{\delta }}_{r}-\dot{\delta }\right)+k\left(\delta_{r}-\delta \right)=f_{\Xi }\\ f_{\Xi }=k_{t}\left(\delta -\delta_{t}\right)+b_{t}\dot{x} \end{array}\right.$$

Equation (38) can be expressed as follows by computing Laplace transforms:

(39) $$\left\{\begin{array}{l} \left(ms^{2}+bs+k\right)\left(\delta_{r}\left(s\right)-\delta \left(s\right)\right)=f_{\Xi }\left(s\right)\\ f_{\Xi }\left(s\right)=\left(b_{t}s+k_{t}\right)\delta \left(s\right)-\frac{k_{t}}{s}\delta_{t} \end{array}\right.$$

According to Equation (39), the planned reference position trajectory can be derived as

(40) $$\delta_{r}\left(s\right)=\frac{ms^{2}+bs+k+b_{t}s+k_{t}}{\left(ms^{2}+bs+k\right)\left(b_{t}s+k_{t}\right)}f_{\Xi }\left(s\right)+\frac{k_{t}\delta_{t}}{s\left(b_{t}s+k_{t}\right)}$$

#### 4.2.2. Inner Loop Trajectory Tracking Control

In position-based impedance control, the force tracking performance depends on the accuracy of the inner loop position control. Here, we use the same robust control strategy as the multirotor platform and define the position error of the manipulator as

(41) $$\vartheta =\eta_{d}-\eta$$

We can design the nonsingular fast terminal sliding mode surface as

(42) $$r=\dot{\vartheta }+\lambda_{1}\vartheta +\rho_{1}diag\left({\left|\vartheta \right|}^{\zeta_{1}/\varsigma_{1}}\right)sgn\left(\vartheta \right)$$

where $\lambda_{1}$ and $\rho_{1}$ are positive definite diagonal matrices, and $\zeta_{1}$ and $\varsigma_{1}$ are positive odd numbers and satisfy $\zeta_{1}<\varsigma_{1}$. Taking the time derivative of Equation (42) results in

(43) $$\begin{array}{cc} B_{\eta }\dot{r} & =B_{\eta }\left(\ddot{\vartheta }+\lambda_{1}\dot{\vartheta }+\frac{\zeta_{1}}{\varsigma_{1}}\rho_{1}diag\left({\left|\vartheta \right|}^{\left(\zeta_{1}-\varsigma_{1}\right)/\varsigma_{1}}\right)diag\left(\dot{\vartheta }\right)sgn\left(\vartheta \right)\right)\\ & =B_{\eta }\left({\ddot{\eta }}_{d}-\ddot{\eta }+\lambda_{1}\dot{\vartheta }+\frac{\zeta_{1}}{\varsigma_{1}}\rho_{1}diag\left({\left|\vartheta \right|}^{\left(\zeta_{1}-\varsigma_{1}\right)/\varsigma_{1}}\right)diag\left(\dot{\vartheta }\right)sgn\left(\vartheta \right)\right)\\ & =B_{\eta }\left({\ddot{\eta }}_{d}+\lambda_{1}\dot{\vartheta }+\frac{\zeta_{1}}{\varsigma_{1}}\rho_{1}diag\left({\left|\vartheta \right|}^{\left(\zeta_{1}-\varsigma_{1}\right)/\varsigma_{1}}\right)diag\left(\dot{\vartheta }\right)sgn\left(\vartheta \right)\right)-B_{\eta }\ddot{\eta }\\ & =B_{\eta }\left({\ddot{\eta }}_{d}+\lambda_{1}\dot{\vartheta }+\frac{\zeta_{1}}{\varsigma_{1}}\rho_{1}diag\left({\left|\vartheta \right|}^{\left(\zeta_{1}-\varsigma_{1}\right)/\varsigma_{1}}\right)diag\left(\dot{\vartheta }\right)sgn\left(\vartheta \right)\right)+H_{\eta }\dot{\eta }+V_{\eta }+F_{c}+F_{d}-F_{\eta }\\ & =B_{\eta }\left({\ddot{\eta }}_{d}-\lambda_{1}\dot{\vartheta }-\frac{\zeta_{1}}{\varsigma_{1}}\rho_{1}diag\left({\left|\vartheta \right|}^{\left(\zeta_{1}-\varsigma_{1}\right)/\varsigma_{1}}\right)diag\left(\dot{\vartheta }\right)sgn\left(\vartheta \right)\right)+H_{\eta }\left({\dot{\eta }}_{d}-\dot{\vartheta }\right)+V_{\eta }+F_{c}+F_{d}-F_{\eta }\\ & =B_{\eta }\left({\ddot{\eta }}_{d}-\lambda_{1}\dot{\vartheta }-\frac{\zeta_{1}}{\varsigma_{1}}\rho_{1}diag\left({\left|\vartheta \right|}^{\left(\zeta_{1}-\varsigma_{1}\right)/\varsigma_{1}}\right)diag\left(\dot{\vartheta }\right)sgn\left(\vartheta \right)\right)\\ & +H_{\eta }\left({\dot{\eta }}_{d}-\lambda_{1}\vartheta -\rho_{1}diag\left({\left|\vartheta \right|}^{\zeta_{1}/\varsigma_{1}}\right)sgn\left(\vartheta \right)\right)+V_{\eta }+F_{c}+F_{d}-F_{\eta }-H_{\eta }r \end{array}$$

Let

(44) $$\Delta \left(\eta_{r}\right)=B_{\eta }{\ddot{\eta }}_{r}+H_{\eta }{\dot{\eta }}_{r}+V_{\eta }+F_{c}$$

where ${\dot{\eta }}_{r}={\dot{\eta }}_{d}-\lambda_{1}\vartheta -\rho_{1}diag\left({\left|\vartheta \right|}^{\zeta_{1}/\varsigma_{1}}\right)sgn\left(\vartheta \right)$, and Equation (43) can be rewritten as

(45) $$B_{\eta }\dot{r}=-H_{\eta }r+\Delta +F_{d}-F_{\eta }$$

Since $\Delta \left(\eta \right)$ contains all model information, the RBF neural network can be used to approximate $\Delta \left(\eta \right)$ to design a robust controller that does not require any model information. We can define the input of RBF neural network as $X={\left(\vartheta \;\dot{\vartheta }\;\eta_{d}\;{\dot{\eta }}_{d}\;{\ddot{\eta }}_{d}\right)}^{T}$; the approximate output of the RBF neural network to the equivalent controller Δ is

(46) $$\hat{\Delta }={\hat{W}}^{T}h\left(X\right)$$

The ideal RBF neural network equivalent control output in Equation (45) is defined as

(47) $$\Delta =W^{T}h\left(X\right)+\varepsilon$$

The estimation error of the neural network is

(48) $$\tilde{\Delta }=\Delta -\hat{\Delta }=W^{T}h\left(X\right)+\varepsilon -{\hat{W}}^{T}h\left(X\right)={\tilde{W}}^{T}h\left(X\right)+\varepsilon$$

We can define the nonsingular global fast terminal sliding mode controller as

(49) $$F_{\eta }=\hat{\Delta }+\lambda_{2}diag\left({\left|r\right|}^{\zeta_{2}}\right)sgn\left(r\right)+\rho_{2}diag\left({\left|r\right|}^{\varsigma_{2}}\right)sgn\left(r\right)+\left(D_{\eta }+\Upsilon_{\eta }\right)sgn\left(r\right)$$

where $\lambda_{2}$ and $\rho_{2}$ are positive definite diagonal matrices, $0<\zeta_{2}<1$ is the ratio of two odd integers, and $\varsigma_{2}>0$. By substituting Equation (49), Equation (45) can be rewritten as

(50) $$\begin{array}{cc} B_{\eta }\dot{r}=-H_{\eta }r\;+ & \Delta +F_{d}-\left(\hat{\Delta }+\lambda_{2}diag\left({\left|r\right|}^{\zeta_{2}}\right)sgn\left(r\right)+\rho_{2}diag\left({\left|r\right|}^{\varsigma_{2}}\right)sgn\left(r\right)+\left(D_{\eta }+\Upsilon_{\eta }\right)sgn\left(r\right)\right)\\ & =-H_{\eta }r+{\tilde{W}}^{T}h+\varepsilon +F_{d}\\ & -\left(\lambda_{2}diag\left({\left|r\right|}^{\zeta_{2}}\right)sgn\left(r\right)+\rho_{2}diag\left({\left|r\right|}^{\varsigma_{2}}\right)sgn\left(r\right)+\left(D_{\eta }+\Upsilon_{\eta }\right)sgn\left(r\right)\right) \end{array}$$

We can define the Lyapunov function as

(51) $$L=\frac{1}{2}r^{T}B_{\eta }r+\frac{1}{2}tr\left({\tilde{W}}^{T}\Gamma^{-1}\tilde{W}\right)$$

Equation (51) can be rewritten as follows by taking the time derivative:

(52) $$\begin{array}{cc} \dot{L} & =r^{T}B_{\eta }\dot{r}+\frac{1}{2}r^{T}{\dot{B}}_{\eta }r+tr\left({\tilde{W}}^{T}\Gamma^{-1}\dot{\tilde{W}}\right)\\ & =r^{T}\left(\begin{array}{c} -H_{\eta }r+{\tilde{W}}^{T}h+\varepsilon +F_{d}-\lambda_{2}diag\left({\left|r\right|}^{\zeta_{2}}\right)sgn\left(r\right)\\ -\rho_{2}diag\left({\left|r\right|}^{\varsigma_{2}}\right)sgn\left(r\right)-\left(D_{\eta }+\Upsilon_{\eta }\right)sgn\left(r\right) \end{array}\right)+\frac{1}{2}r^{T}{\dot{B}}_{\eta }r+tr\left({\tilde{W}}^{T}\Gamma^{-1}\dot{\tilde{W}}\right)\\ & =-\sum_{i=1}^{n}\lambda_{2i}{\left|r_{i}\right|}^{\zeta_{2}+1}-\sum_{i=1}^{n}\rho_{2i}{\left|r_{i}\right|}^{\varsigma_{2}+1}+\frac{1}{2}r^{T}\left({\dot{B}}_{\eta }-2H_{\eta }\right)r-tr{\tilde{W}}^{T}\left(\Gamma^{-1}\dot{\hat{W}}-hr^{T}\right)\\ & +r^{T}\left(\varepsilon +F_{d}\right)-{\left|r\right|}^{T}\left(D_{\eta }+\Upsilon_{\eta }\right) \end{array}$$

The manipulator dynamic model parameters are skewed symmetrically, so

(53) $$r^{T}\left({\dot{B}}_{\eta }-2H_{\eta }\right)r=0$$

We can define RBF neural network adaptive law as

(54) $$\dot{\hat{W}}=\Gamma hr^{T}$$

Equation (52) can be written as follows by substituting Equations (53) and (54):

(55) $$\dot{L}=-\sum_{i=1}^{n}\lambda_{2i}{\left|r_{i}\right|}^{\zeta_{2}+1}-\sum_{i=1}^{n}\rho_{2i}{\left|r_{i}\right|}^{\varsigma_{2}+1}+r^{T}\left(\varepsilon +F_{d}\right)-{\left|r\right|}^{T}\left(D_{\eta }+\Upsilon_{\eta }\right)$$

**Figure 4:**
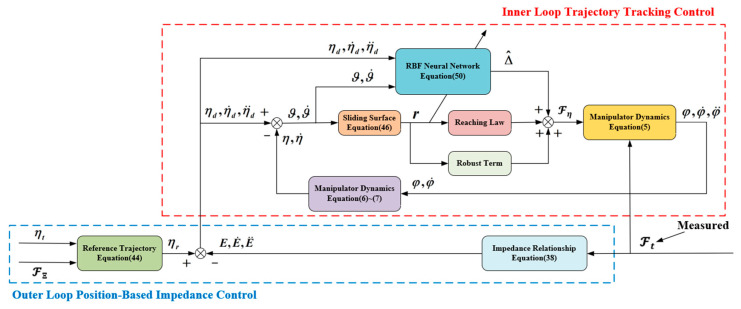
The structure of the manipulator control system.

When $D_{\eta }\geq \left|F_{d}\right|$ and $\Upsilon_{\eta }\geq \left|\varepsilon \right|$, $\dot{L}\leq 0$, the closed-loop system is stable. Based on the above analysis, the structure of the manipulator control system is shown in Figure 4.

## 5. Simulations

In this section, some simulations are performed on the aerial manipulator to verify the effectiveness of the proposed control strategy. These simulations for the quadrotor and manipulator were performed separately in MATLAB/Simulink R2022a software. Since many studies have been completed on ideal conditions, this study mainly works on the situation with random interference.

### 5.1. Simulation Setting

The aerial manipulator used in the simulation is a quadrotor equipped with a planar 3DOF manipulator. The parameters of the aerial manipulator are m=2.32 kg, $g=9.8\;m\cdot s^{-2}$, $\left[\begin{array}{ccc} I_{xx} & I_{xx} & I_{xx} \end{array}\right]=\left[\begin{array}{ccc} 0.032 & 0.032 & 0.048 \end{array}\right]\;kg\cdot m^{2}$, l=0.55 m, $\left[\begin{array}{ccc} m_{1} & m_{2} & m_{3} \end{array}\right]=\left[\begin{array}{ccc} 0.26 & 0.26 & 0.18 \end{array}\right]\;kg$, $\left[\begin{array}{ccc} l_{1} & l_{2} & l_{3} \end{array}\right]=\left[\begin{array}{ccc} 0.25 & 0.25 & 0.4 \end{array}\right]\;m$. The gains of each subsystem controller of the aerial manipulator are shown in Table 1.

In many papers, NGFTSMC without neural networks has been proven to have sound control effects for multi-rotors and manipulators, so the simulation in this study only compares NGFTSMC with and without neural networks. The selection of all parameters was obtained through multiple simulations and can improve the system’s performance. To test the robustness of the controller, the system disturbance was set as a random disturbance within ±5% of the nominal model of the system.

### 5.2. Simulation Results and Analysis

#### 5.2.1. Quadrotor Platform Subsystem

The initial position and Euler angle of the quadrotor subsystem are $\left[\begin{array}{ccc} 0 & 0 & 0 \end{array}\right]$ m and $\left[\begin{array}{ccc} 0 & 0 & 0 \end{array}\right]$ rad, and the desired position and Euler angle are set to $\left[\begin{array}{ccc} 2 & 2 & 4 \end{array}\right]$ m and $\left[\begin{array}{ccc} 0 & 0 & 0 \end{array}\right]$ rad. In 0 to 5 s, the quadrotor flies to the destination and remains hovering after 5 s. The manipulator moves to the target in 5 to 10 s and gradually forms a contact force 5 N with the target in 10 to 15 s. In 15 to 20 s, the contact force remains unchanged. Since there is force contact between the manipulator and the target from 10 to 20 s, the desired pitch angle of the quadrotor can be calculated by Equation (12) and the desired contact force.

**Figure 5:**
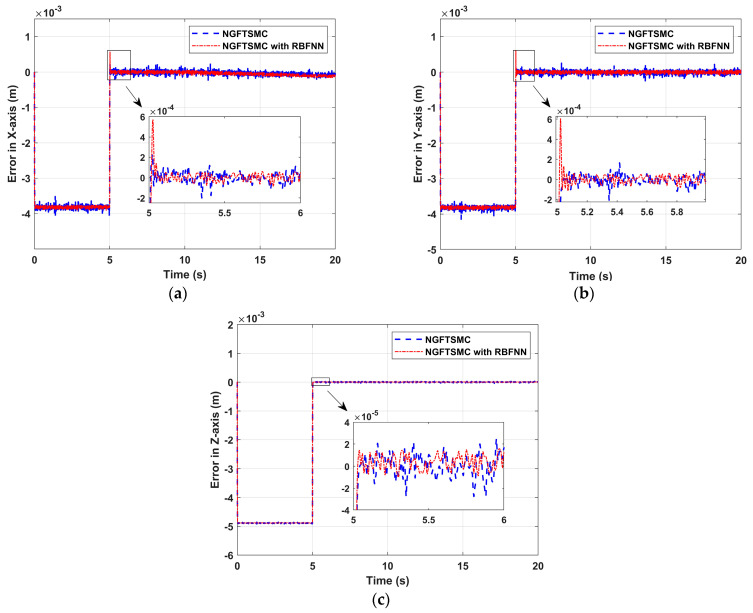
The position tracking performance of the quadrotor platform: (**a**) Error in X-axis; (**b**) Error in Y-axis; (**c**) Error in Z-axis.

Figure 5 shows the tracking error of the quadrotor platform in the X-Y-Z axis. The system states controlled by both controllers can track the desired trajectory, but the performance on the X-Y axis is slightly different from that on the Z-axis. In Figure 5a,b, the error of the system state controlled by NGFTSMC based on RBFNN is about 70% that of regular NGFTSMC. Nevertheless, there is a particular time point that at 5 s, the system state error of NGFTSMC control based on RBFNN has a significant overshoot. The overshoot value on the X-axis is 5.7 × 10^−4^, and the overshoot value on the Y-axis is 5.8 × 10^−4^. At the same time, the error controlled by regular NGFTSMC has no obvious abnormality change at 5 s. In Figure 5c, the performance of both controllers on the Z-axis is more stable. The error of the system state controlled by RBFNN-based NGFTSMC is 30% that of regular NGFTSMC, and there is no overshoot in both performances.

**Figure 6:**
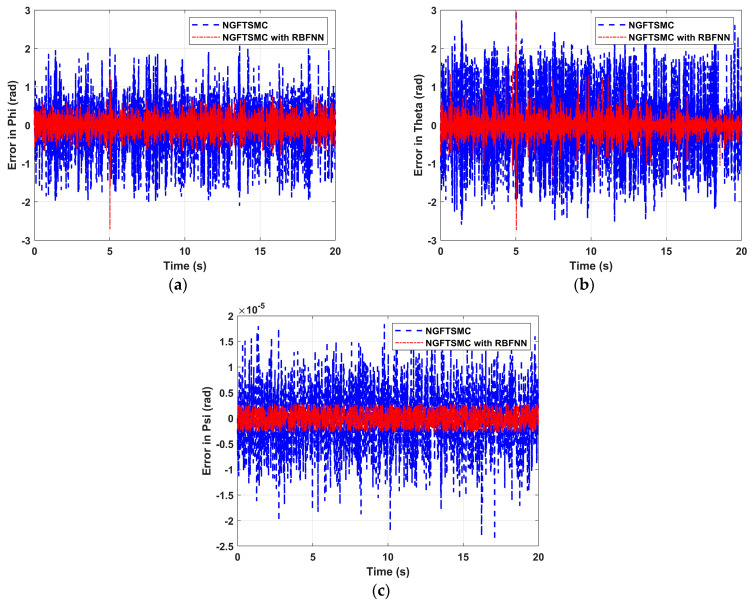
The attitude tracking performance of the quadrotor platform: (**a**) Error in Phi; (**b**) Error in Theta; (**c**) Error in Psi.

Figure 6 depicts the variation of the system Euler angle tracking error over time. As shown in Figure 6a,b, the overshoot of the state error controlled by NGFTSMC based on RBFNN is about 25% that of the regular NGFTSMC. Moreover, corresponding to Figure 5a,b, the state error controlled by the proposed NGFTSMC also has a remarkable overshoot at 5 s. This is because the roll angle and pitch angle are coupled with the motion system in the Y-axis and X-axis, respectively, so their error shift patterns should be similar to the error trajectories in the Y-axis and X-axis, respectively. The yaw angle is not coupled with other states, so its control accuracy is better than the others. As shown in Figure 6c, the yaw error of the NGFTSMC-controlled system based on RBFNN is 0.3 × 10^−5^, which is only about 20% of the regular NGFTSMC system.

**Figure 7:**
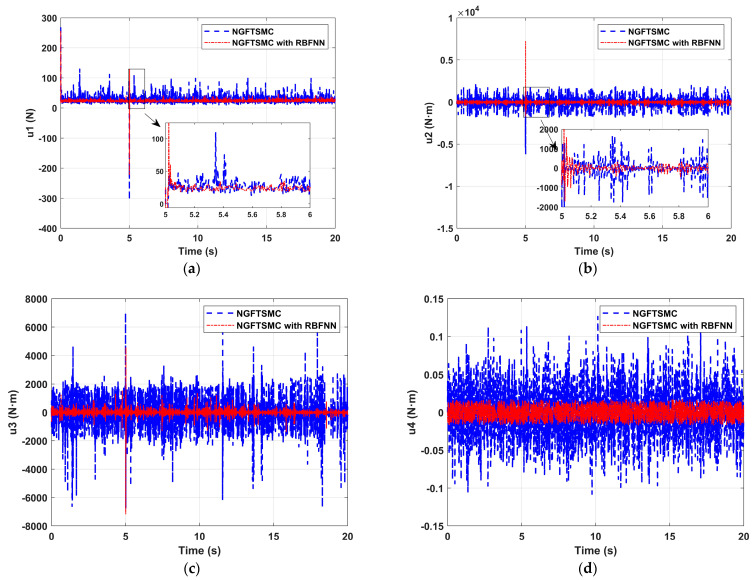
The control input performance of the quadrotor platform: (**a**) $u_{1}$; (**b**) $u_{2}$; (**c**) $u_{3}$; (**d**) $u_{4}$.

Figure 7 depicts the variation in the output torque of the quadrotor platform over time. It can be seen that the torques controlled by NGFTSMC based on RBFNN are smaller than those of the regular NGFTSMC, which means the rotation speed of the motor is slower. Decreasing rotational speed means the motor is more accessible to implement and mechanical loss is less, which is crucial for a mechanical system.

#### 5.2.2. Manipulator Subsystem

The initial state of the manipulator subsystem is $\left[0\;0\;0\right]$ rad. According to the target position and inverse kinematics, the desired state of the manipulator can be calculated as $\left[0.46\;0.79\;0.31\right]$ rad. First, the manipulator moves from the initial posture to the desired posture in 0 to 5 s. Then, the manipulator gradually forms a contact force 5 N with the target in 5 to 10 s. Finally, the contact force is maintained for 10 to 15 s.

**Figure 8:**
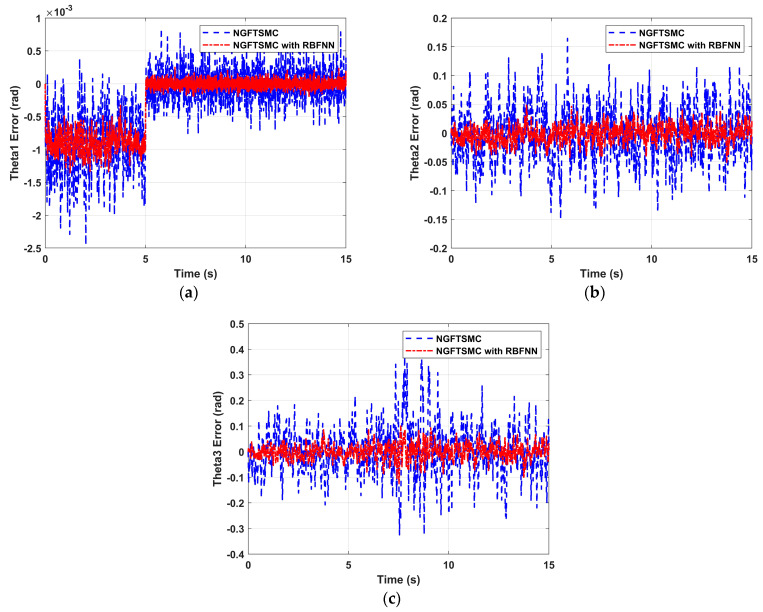
The trajectory tracking performance of the manipulator: (**a**) The tracking error of joint 1; (**b**) The tracking error of joint 2; (**c**) The tracking error of joint 3.

Figure 8 illustrates the tracking error results of the manipulator joints 1, 2, and 3 moving along the desired trajectory. It can be seen in the figure that the errors controlled by the NGFTSMC based on RBFNN are more miniature than those of the regular NGFTSMC, with a general error of about 50% and a peak error of less than 30%. Meanwhile, the manipulator is connected in a series, and the error of the joint closer to the manipulator base is minor. According to the analysis of system operation, this is consistent with the situation of the actual system.

**Figure 9:**
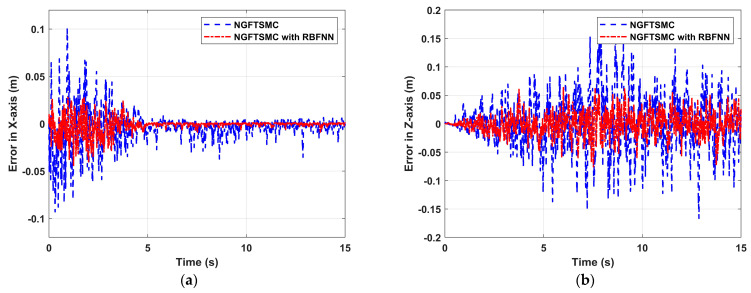
The trajectory tracking performance of the end of manipulator: (**a**) Error in X-axis; (**b**) Error in Z-axis.

Figure 9 depicts the variation in the position error of the manipulator with time. In Figure 9a, the state error of the approaching phase is more considerable than that of the contact phase, and the error of GFTSMC based on RBFNN is about 30% that of regular GFTSMC. The contact force only exists on the X-axis, so after contacting the target, the smaller error in the X-axis means more accurate force tracking. In Figure 9b, the error gradually increases from 0 to 5 s, while the error is relatively stable within 5 to 15 s, and the error of NGFTSMC based on RBFNN is about 50% that of regular GFTSMC. Since the manipulator has no contact force in the Z-axis and is not constrained, it can be seen that the state error in the Z-axis is significantly greater than the state error in the X-axis.

**Figure 10:**
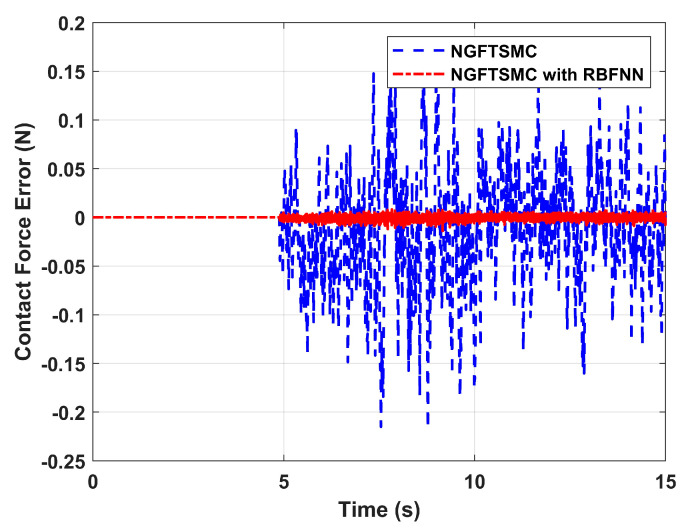
The contact force error between the manipulator and the target.

Figure 10 portrays the contact force error between the manipulator and the target over time. It can be seen that during the contact process of 5 to 15 s, the force-tracking capability of the NGFTSMC based on RBFNN is significantly better than that of the regular NGFTSMC, and the error is about 0.02 N, which is consistent with the performance of the position error in the X-axis. Therefore, the proposed control scheme has better tracking performance and is more effective than regular NGFTSMC.

## 6. Experiments

**Figure 11:**
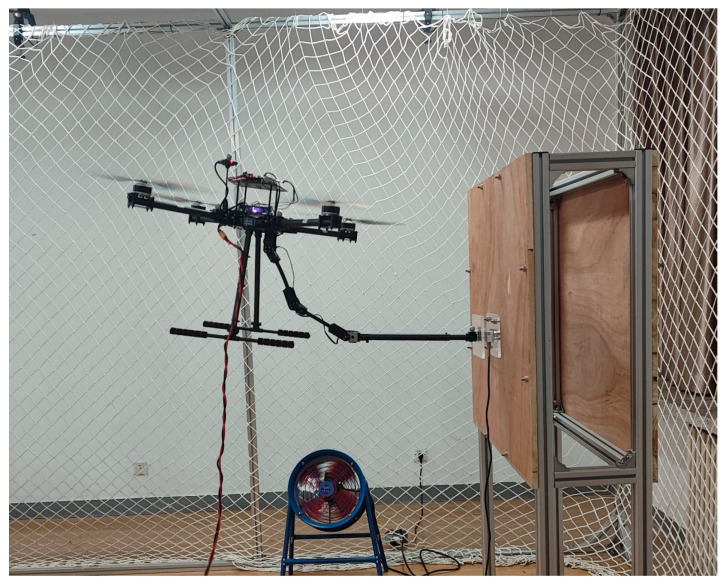
The photograph of the contact force experiment.

In this section, some experiments are accomplished to verify the effectiveness of the proposed controller in exerting precise contact force on a target using the aerial manipulator. The quadrotor base of the aerial manipulator used in the experiment is a 680 mm diameter quadrotor equipped with four 15-inch propellers and a Pixhawk autopilot for low-level driver control. The manipulator is a planar three-link arm installed at the bottom of the quadrotor base. The control algorithms for the quadrotor and manipulator are executed on a Raspberry Pi 4b onboard computer. In addition, a motion-capture system and a pressure-detection system are used to measure the position, attitude, and contact force of the aerial manipulation system precisely. The photograph of the contact force experiment is pictured in Figure 11.

**Figure 12:**
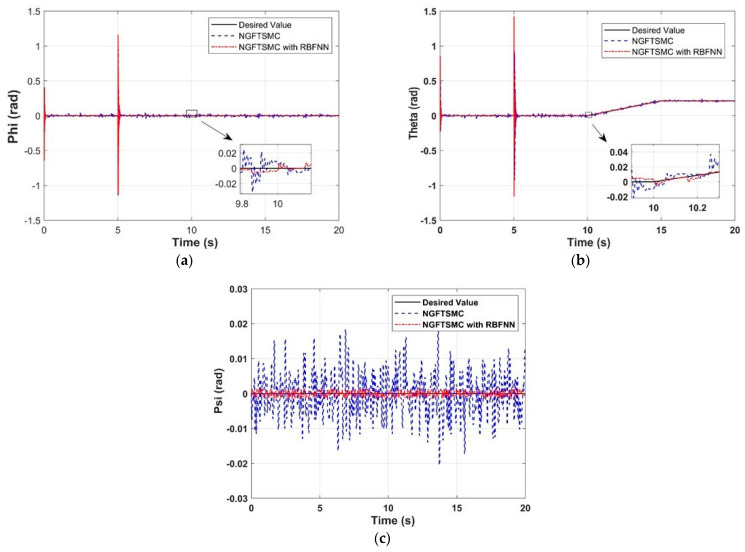
Euler angles of the quadrotor platform in the experiment: (**a**) The experimental results in Phi; (**b**) The experimental results in Theta; (**c**) The experimental results in Psi.

**Figure 13:**
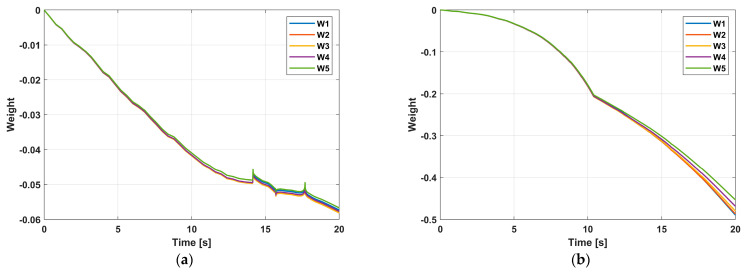
Weights of the RBFNN: (**a**) Quadrotor; (**b**) Manipulator.

Figure 12 exhibits the variation in Euler angles of the quadrotor platform over time in the experiment. It can be noticed that both controllers can quickly converge the system state to the desired trajectory. However, the state vector fluctuations of the system controlled by NGFTSMC based on RBFNN are minor compared to regular NGFTSMC, where the roll angle and pitch angle are 30%, and the yaw angle is 10%. The influence of external disturbances is not obvious in the laboratory conditions, which would be the reason that the controller performs very well. In addition, there were notable oscillations in roll and pitch at the 5th second. This is because the manipulator generates a large counter-torque at the moment of operation, which dramatically impacts the stability of the quadrotor platform. However, the rapid convergence of the controller allowed the quadrotor platform to quickly restore balance without affecting the operation of the manipulator. Moreover, weight convergence can be observed in Figure 13.

**Figure 14:**
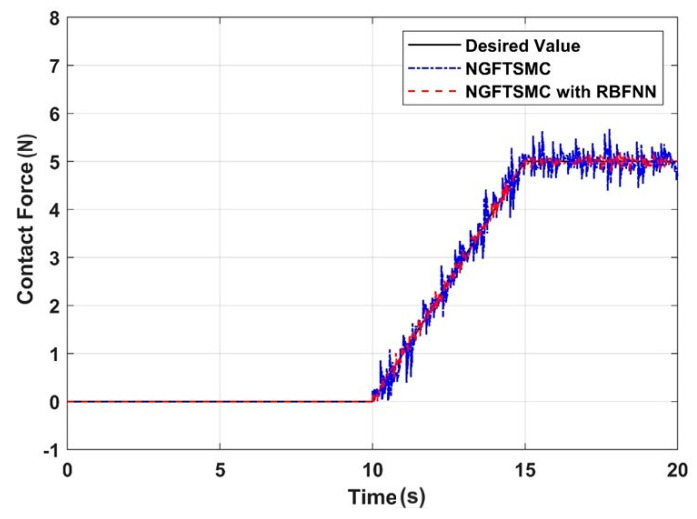
The contact force between the aerial manipulator and the target.

**Figure 15:**
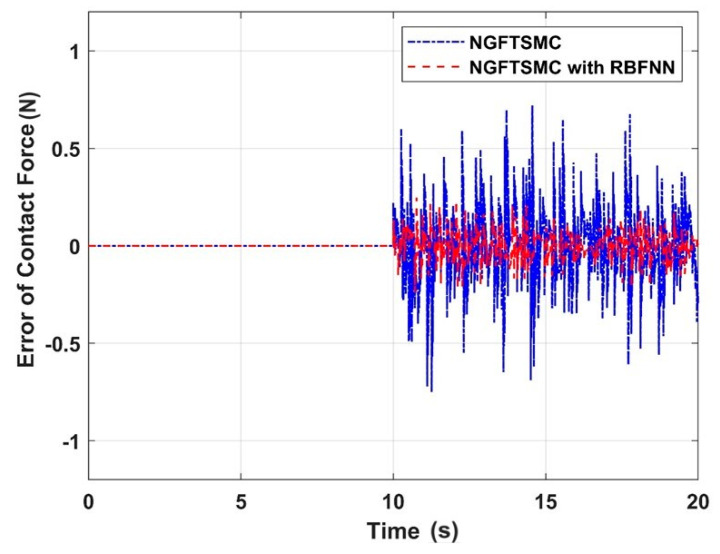
The error of contact force between the aerial manipulator and the target.

Figure 14 illustrates the contact force between the aerial manipulator and the target measured by the pressure detection system, and Figure 15 depicts the error between the measured and expected contact forces. It can be concluded that in the control system of NGFTSMC based on RBFNN, the contact force error can be controlled within 0.25 N, which is 30% of that of the regular NGFTSMC-controlled system. The results indicate that the proposed control scheme is effective in achieving precise contact force between the aerial manipulator and the target. Through analysis, it can be understood that the stronger the anti-interference ability of the quadrotor platform, the higher the accuracy of the contact force between the aerial manipulator and the target.

## 7. Conclusions

This study analyzes the characteristics of aerial contact operations and proposes a new control strategy for the aerial manipulation system. The multirotor and manipulator are controlled separately as two independent subsystems of the aerial manipulator. The multirotor subsystem exclusively serves as an aerial platform for the manipulator, providing a stable base in the air. Meanwhile, the manipulator subsystem merely performs contact force operations as an instrument. The interactions between both subsystems are regarded as mutual disturbances. First, each subsystem is modeled separately, and the mutual effects are analyzed and expressed using kinematic methods. Then, a robust sliding mode controller is developed for the aerial platform subsystem, and an adaptive RBF neural network controller is designed to eliminate the dependence on the system model through online learning. In addition, a double-loop impedance position controller is devised for the manipulator to execute compliant control of the contact force between the manipulator and the target. The simulation results indicate that the proposed control strategy has good trajectory-tracking capabilities and can accurately control the contact force. Finally, the experiment verified the effectiveness of the proposed control method, showing that the contact force error between the aerial manipulator and the target can be controlled within 0.25 N, which is 30% of the error of the comparative control scheme.

In this work, the impedance parameters of the target are known, but in unknown en-vironments, the impedance parameters of the target cannot be obtained directly. Future work will focus on using the learning method to generate the desired position only by the desired force and minimize the position error without knowing the environment or the impedance parameters.

## Figures and Tables

**Table 1:** The gains of each subsystem controller of the aerial manipulator.

Subsystem	Parameters	Values
Quadrotor	$\alpha_{1},\beta_{1},\mu_{1},\nu_{1}$	200, 20, 3, 5
	$\alpha_{2},\beta_{2},\mu_{2},\nu_{2}$	600, 200, 3, 5
	c	[−2 −1 0 1 2]
	b	5
	W	[0 0 0 0 0]
	ξ	0.4
Manipulator	m,b,k	1, 500, 1000
	$k_{t},b_{t}$	10,000, 10
	$\lambda_{1},\rho_{1},\zeta_{1}\varsigma_{1}$	100I,20I,3,5
	$\lambda_{2},\rho_{2},\zeta_{2},\varsigma_{2}$	20I,20I,3,5
	Γ	0.01I

## Data Availability

The data used to support the findings of this study are included within the article.
